# Supplementary material for: Whole-body analysis of TRPML3 (MCOLN3) expression using a GFP-reporter mouse model reveals widespread expression in secretory cells and endocrine glands
Source: PLoS One. 2022 Dec 15;17(12):e0278848. doi: 10.1371/journal.pone.0278848 (PMC10045552; doi:10.1371/journal.pone.0278848)
Supplement: S1 File — (PDF) [file pone.0278848.s001.pdf]

Supporting Information

Whole-body analysis of TRPML3 expression using a GFP-reporter mouse model reveals widespread expression in secretory cells and endocrine glands

Barbara Spix[1¶], Andrew J. Castiglioni[2¶], Natalie N. Remis[2,3¶], Emma N. Flores[2,4], Philipp Wartenberg[5], Amanda Wyatt[5], Ulrich Boehm[5], Thomas Gudermann[1], Martin Biel[6], Jaime García-Añoveros[2,3,4,7]*, Christian Grimm[1]*

Supplementary Figures

Figure S1. In situ on P2 *Trpml3*$^{+/+}$ kidney with 3' *Trpml3* probe.

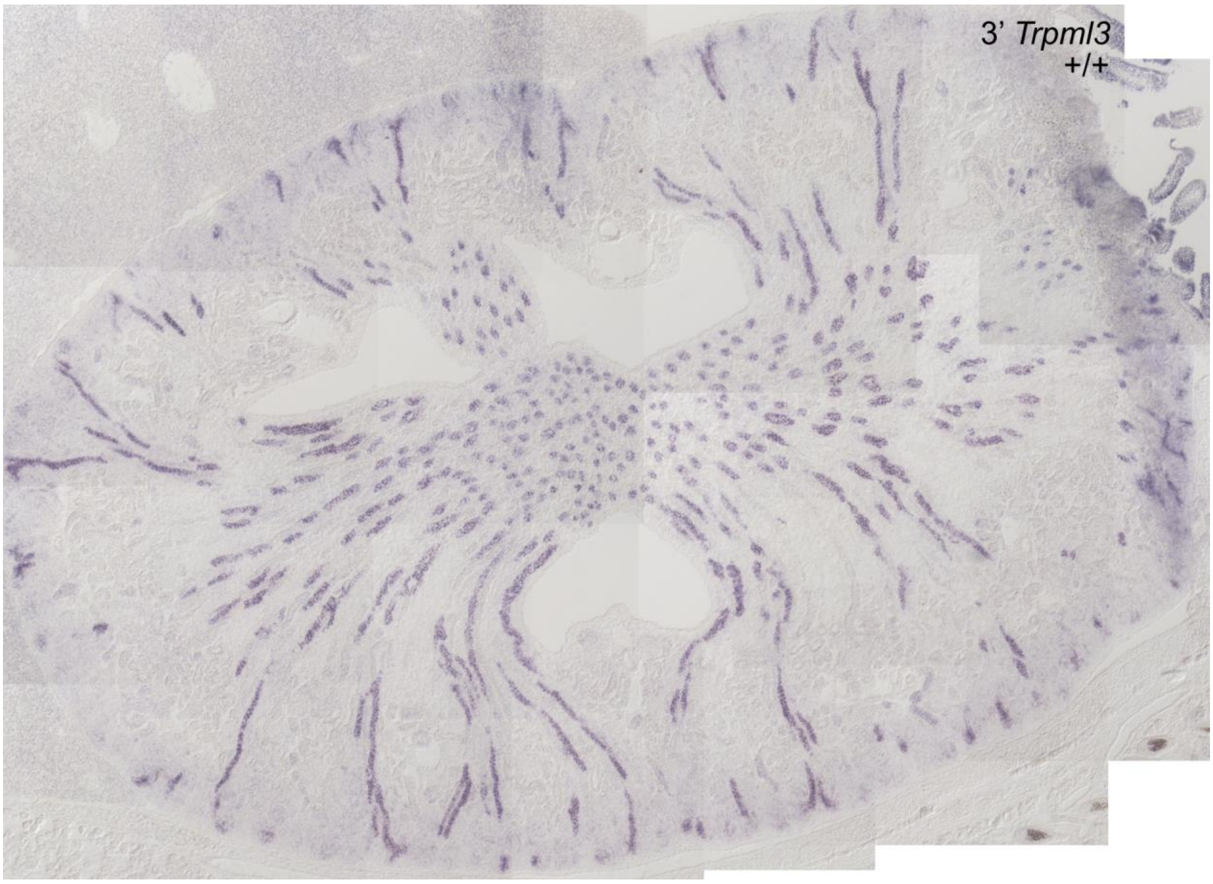

Figure S1. In situ on P2 *Trpml3*$^{+/+}$ kidney with 3' *Trpml3* probe.
