# Supplementary material for: Whole-body analysis of TRPML3 (MCOLN3) expression using a GFP-reporter mouse model reveals widespread expression in secretory cells and endocrine glands
Source: PLoS One. 2022 Dec 15;17(12):e0278848. doi: 10.1371/journal.pone.0278848 (PMC10045552; doi:10.1371/journal.pone.0278848)
Supplement: Attachment — Submitted filename: PONE-D-22-24077-PbyPresponse letter.pdf [file pone.0278848.s002.pdf]

PONE-D-22-24077
Whole-body analysis of TRPML3 expression using a GFP-reporter mouse model reveals widespread expression in secretory cells and endocrine glands
PLOS ONE

Dear Dr. Grimm,

Thank you for submitting your manuscript to PLOS ONE. After careful consideration, we feel that it has merit but does not fully meet PLOS ONE's publication criteria as it currently stands. Therefore, we invite you to submit a revised version of the manuscript that addresses the concerns of the reviewers.

Please submit your revised manuscript by Dec 03 2022 11:59PM. If you will need more time than this to complete your revisions, please reply to this message or contact the journal office at plosone@plos.org. When you're ready to submit your revision, log on to https://www.editorialmanager.com/pone/ and select the 'Submissions Needing Revision' folder to locate your manuscript file.

Please include the following items when submitting your revised manuscript:

- A rebuttal letter that responds to each point raised by the reviewers. You should upload this letter as a separate file labeled 'Response to Reviewers'.
- A marked-up copy of your manuscript that highlights changes made to the original version. You should upload this as a separate file labeled 'Revised Manuscript with Track Changes'.
- An unmarked version of your revised paper without tracked changes. You should upload this as a separate file labeled 'Manuscript'.

If you would like to make changes to your financial disclosure, please include your updated statement in your cover letter. Guidelines for resubmitting your figure files are available below the reviewer comments at the end of this letter.

If applicable, we recommend that you deposit your laboratory protocols in protocols.io to enhance the reproducibility of your results. Protocols.io assigns your protocol its own identifier (DOI) so that it can be cited independently in the future. For instructions see: https://journals.plos.org/plosone/s/submission-guidelines#loc-laboratory-protocols. Additionally, PLOS ONE offers an option for publishing peer-reviewed Lab Protocol articles, which describe protocols hosted on protocols.io. Read more information on sharing protocols at https://plos.org/protocols?utm_medium=editorial-email&utm_source=authorletters&utm_campaign=protocols.

We look forward to receiving your revised manuscript.

Kind regards,

Alexander G. Obukhov, Ph.D.
Academic Editor
PLOS ONE

Journal Requirements:

When submitting your revision, we need you to address these additional requirements.

1. Please ensure that your manuscript meets PLOS ONE's style requirements, including those for file naming. The PLOS ONE style templates can be found at https://journals.plos.org/plosone/s/file?id=wjVg/PLOSOne_formatting_sample_main_body.pdf and https://journals.plos.org/plosone/s/file?id=ba62/PLOSOne_formatting_sample_title_authors_affiliations.pdf

Authors: Checked

2. To comply with PLOS ONE submissions requirements, in your Methods section, please provide additional information regarding the experiments involving animals and ensure you have included details on (1) methods of sacrifice, (2) methods of anesthesia and/or analgesia, and (3) efforts to alleviate suffering.

Authors: Done

3. Thank you for stating the following in the Acknowledgments Section of your manuscript:

"Financial Support: RO1 DK111032 and R01 DC015903 (to JGA), T32 NRSA NS041234 (to AJC and ENF), F31 NRSA DC010529 (to NNR), German Research Foundation (GRK2338 P08 to CG and MB, P09 to TG, SFB/TRR152 Z02 to UB, P04 to CG, P12 to MB, P15 and the German Center of Lung Research, DZL, to TG)."

We note that you have provided funding information that is not currently declared in your Funding Statement. However, funding information should not appear in the Acknowledgments section or other areas of your manuscript. We will only publish funding information present in the Funding Statement section of the online submission form.
Please remove any funding-related text from the manuscript and let us know how you would like to update your Funding Statement. Currently, your Funding Statement reads as follows:

"Financial Support: RO1 DK111032 and R01 DC015903 (to JGA), T32 NRSA NS041234 (to AJC and ENF), F31 NRSA DC010529 (to NNR), German Research Foundation (GRK2338 P08 to CG and MB, P09 to TG, SFB/TRR152 Z02 to UB, P04 to CG, P12 to MB, P15 and the German Center of Lung Research, DZL, to TG).

The funders had no role in study design, data collection and analysis, decision to publish, or preparation of the manuscript."

Please include your amended statements within your cover letter; we will change the online submission form on your behalf.

Authors: Please update the online form according to the indicated funders above. Thanks.

4. We note that you have included the phrase "data not shown" in your manuscript. Unfortunately, this does not meet our data sharing requirements. PLOS does not permit references to inaccessible data. We require that authors provide all relevant data within the paper, Supporting Information files, or in an acceptable, public repository. Please add a citation to support this phrase or upload the data that corresponds with these findings to a stable repository (such as Figshare or Dryad) and provide and URLs, DOIs, or accession

numbers that may be used to access these data. Or, if the data are not a core part of the research being presented in your study, we ask that you remove the phrase that refers to these data.

Authors: Removed

5. Please review your reference list to ensure that it is complete and correct. If you have cited papers that have been retracted, please include the rationale for doing so in the manuscript text, or remove these references and replace them with relevant current references. Any changes to the reference list should be mentioned in the rebuttal letter that accompanies your revised manuscript. If you need to cite a retracted article, indicate the article's retracted status in the References list and also include a citation and full reference for the retraction notice.

Authors: Done

[Note: HTML markup is below. Please do not edit.]

Reviewers' comments:

Reviewer's Responses to Questions

Comments to the Author

1. Is the manuscript technically sound, and do the data support the conclusions?

The manuscript must describe a technically sound piece of scientific research with data that supports the conclusions. Experiments must have been conducted rigorously, with appropriate controls, replication, and sample sizes. The conclusions must be drawn appropriately based on the data presented.

Reviewer #1: Yes

Reviewer #2: Yes

---

2. Has the statistical analysis been performed appropriately and rigorously?

Reviewer #1: Yes

Reviewer #2: N/A

---

3. Have the authors made all data underlying the findings in their manuscript fully available?

The PLOS Data policy requires authors to make all data underlying the findings described in their manuscript fully available without restriction, with rare exception (please refer to the Data Availability Statement in the manuscript PDF file). The data should be provided as part of the manuscript or its supporting information, or deposited to a public repository. For example, in addition to summary statistics, the data points behind means, medians and

variance measures should be available. If there are restrictions on publicly sharing data—e.g. participant privacy or use of data from a third party—those must be specified.

Reviewer #1: Yes

Reviewer #2: Yes

---

4. Is the manuscript presented in an intelligible fashion and written in standard English?

PLOS ONE does not copyedit accepted manuscripts, so the language in submitted articles must be clear, correct, and unambiguous. Any typographical or grammatical errors should be corrected at revision, so please note any specific errors here.

Reviewer #1: Yes

Reviewer #2: Yes

---

5. Review Comments to the Author

Please use the space provided to explain your answers to the questions above. You may also include additional comments for the author, including concerns about dual publication, research ethics, or publication ethics. (Please upload your review as an attachment if it exceeds 20,000 characters)

Reviewer #1: This study systematically applied several methodological approaches to validate the expression patterns of the endolysosomal TRP channel, TRPML3, in various mouse tissues. The authors firstly confirmed the presence of TRPML3 in lung alveolar macrophages, olfactory sensory neurons, skin melanocytes, and principle cells of the collecting duct of the kidney. Secondly, the authors made new discovery on that TRPML3 is also expressed in thyroid/parathyroid, salivary, adrenal and pituitary gland. The confocal images shown in the study are clear and conclusive.

On the whole, this work provides the basic knowledge on the expression pattern of TRPML3 throughout the mouse body, which should be highly appreciated.

Authors: We thank the reviewer for his positive comments. We have addressed his suggestions as outlined below.

To further improve this manuscript, the following minor concerns should be addressed:

1. The statistical columns (Fig. 1A, 2C and 4B) lack of error bars.

Authors: 1. This is because these RT-qPCRs were done each from a single source (organ for one animal). We did them in triplicate, as is customary for RT-qPCR in order to confirm the results are reproducible, but adding error bars would not make statistical sense. Accordingly, we did not make any statistical analyses from these graphs. We used them as a first pass indicator of which organs and at what stages may express Trpml3, and then proceed to confirm these results by in situ hybridization probes and immunohistochemistry in many samples of the relevant organ (in addition to the Trpml3IRES-Cre/eR26-τGFP reporter mice). We are attaching the excel spreadsheet with the data for these three RT-qPCR graphs. We could add error bars by separately calculating the data for each replicate, but these are not biological replicates and hence this would be IOO misleading.

2. In this study, two antisera raised against different regions of TRPML3 (NT and CT1) were used for detecting TRPML3 expression. However, the CT1 antiserum was adopted only once when examining adult lungs (Fig. 2), while NT antiserum appeared more frequent application. Is there any reason for the preference?

Authors: The only reason for using more often the NT antiserum is availability, as it is commercially available, whereas the CT1 antiserum is a gift from Markus Delling and David Clapham and, as such, we have limited amounts of it. However, we have used both antibodies in multiple tissues in addition to lung, such as cochlea (hair cells and principal cells of the stria vascularis), olfactory epithelium and vomeronasal organ chemosensory neurons (Castiglioni et al., 2011. J Comp Neurol).

3. How to explain the observation that only a subset of NT antiserum immunoreactivities was removed from the Trpml3-/- kidney?

Authors: The immunoreactivities that remain in the Trpml3 KOs are non-specific (cross reacting with proteins other than TRPML3). We have seen this in other organs, in which an antibody labels a cell type in the KO (hence in the absence of TRPML3) and that cell type does not express Trpml3 mRNA as determined by in situ hybridization.

We have clarified this by modifying the following statement: "Our NT antisera immunoreacted with numerous tubes in the Trpml3+/+ kidney, but only a subset of these immunoreactivities were removed in the Trpml3-/- kidney. In prior analyses, we have seen certain immunoreactivities in other tissues that were not removed by the Trpml3-/- (30) and therefore we focused our attention on tubes whose NT immunoreactivities were removed in Trpml3-/- tissue and specific to TRPML3 (Fig. 5G, I)". Instead, we now simply state that: "Our NT antisera immunoreacted with numerous tubes in the Trpml3+/+ kidney, but only a subset of these immunoreactivities was removed in the Trpml3-/- kidney. In prior analyses, we have seen certain immunoreactivities in other tissues that were not removed by the Trpml3-/- (30) and concluded they were non-specific (detecting a protein other than TRPML3). Therefore, we focused our attention on tubes whose NT immunoreactivities were removed in Trpml3-/- tissue, which are specific to TRPML3 (Fig. 5G, I)".

4. Trpml3 mRNA expression of B cells in thymus was analyzed. How about plasma cells?

Authors: We did not examine plasma cells, only solid tissues.

Reviewer #2: The paper is methodologically sound and contains large amount of data that can be useful for researchers interested in various tissues. Below are listed some comments.

1) The official name of the gene is Mcoln3 (https://www.ncbi.nlm.nih.gov/gene/171166). Some researchers may prefer the name TRPML3 but more and more researchers will search for the official name. Therefore, add in parentheses the official name of the gene in the title of the paper. It is an easy way to increase the impact of the paper.

Authors: We thank the reviewer for his comment and have added Mcoln3 as recommended.

2) In addition to papers specifically studying TRPML3 (Mcoln3) there are also online databases showing pattern of expression of various genes. Two databases containing information about expression of Mcoln3 are:
http://mouse.brain-map.org/gene/show/82359
http://mousebrain.org/adolescent/genesearch.html
Please, include these databases in discussion (they should be cited together with papers that for the first time described these databases (https://doi.org/10.1038/nature05453 and https://doi.org/10.1016/j.cell.2018.06.021)

Authors: This has been added accordingly.

3) It is not clear how many mice were used to obtain the data. This information can be provided in figure descriptions (separately for each method).

Authors: This info is now provided in the M&M section.

---

6. PLOS authors have the option to publish the peer review history of their article (what does this mean?). If published, this will include your full peer review and any attached files.

If you choose "no", your identity will remain anonymous but your review may still be made public.

Do you want your identity to be public for this peer review? For information about this choice, including consent withdrawal, please see our Privacy Policy.

Reviewer #1: **Yes:** Wuyang Wang

Reviewer #2: No
